# Impact of COVID-19 on emergency department use among home care recipients: Alberto Peano

**DOI:** 10.1093/eurpub/ckac129.253

**Published:** 2022-10-25

**Authors:** A Peano, E Minutiello, G Politano, M Dalmasso, MM Gianino

**Affiliations:** 1 Department of Sciences of Public Health and Pediatrics, University of Turin, Turin, Italy; 2 Unit of Epidemiology, Local Health Unit TO3, Grugliasco, Italy; 3 Department of Control and Computer Engineering, The Polytechnic University of Turin, Turin, Italy

## Abstract

**Background:**

The impact of COVID-19 pandemic on Emergency Department (ED) was remarkable throughout Europe. We focused upon ED utilization among integrated home care (IHC) recipients comparing ED between pandemic period with pre-pandemic (February -December 2020 and 2019, respectively) in Piedmont, Italy.

**Methods:**

A retrospective observational study was conducted. All recipients of IHC during the two periods studied were enrolled and all ED visits that occurred among IHC recipients were accounted for. Several variables related to IHC admission, reason of ED visits and demographic characteristics were collected. The average of ED visits in pre-pandemic and pandemic periods were calculated. Analyses were stratified by all variables.

**Results:**

Patients enrolled were 11968 in 2019 and 8938 in 2020. In 2019, 3573 patients had at least one ED visit and 1668 patients in 2020. Number of ED visits was 5503 in 2019 and 2197 in 2020. The average of ED visits in 2020 has reduced in comparison with 2019 (0.464 C.I. [0.44-0.489] and 0.24 C.I. [0.227-0.252], p < 0.001 in 2019 and 2020 respectively). This reduction is regardless of sex, age, duration of IHC, presence of a non-family caregiver or reason for ED visits, except for abdominal pain, cardiac rhythm alteration and gynaecological symptoms. The averages of ED visits were significantly lower for IHC recipients with neoplasm (0.549 C.I. [0.513-0.585] and 0.328 C.I. [0.298-0.358], p < 0.001, and with low level of emergency (1.77 C.I. [1.662-1.877] and 1.397 C.I. [1.348-1.447], p < 0.036), but an increase in mortality rate was not registered.

**Conclusions:**

Our results showed a reduction of ED visits among integrated home care recipients in pandemic period in comparison with pre-pandemic period. If the reduction can be the consequence of an unprepared health service that needs of necessary changes in its organization, these results suggest a great potential of the home care system to reduce the use of the hospital especially for low-risk conditions.

**Key messages:**

• The COVID-19 pandemic overwhelmed health services of all European Countries. A reduced utilization of ED has been shown by literature, especially during the early phase of the COVID-19 pandemic.

• We showed a reduction in IHC recipients and a great decrease in ED visits among IHC patients in 2020 versus 2019, mainly in oncological patients, while an increase in mortality rate was not reported.

